# Augmentation of CD47/SIRP**α** signaling protects cones in genetic models of retinal degeneration

**DOI:** 10.1172/jci.insight.150796

**Published:** 2021-08-23

**Authors:** Sean K. Wang, Yunlu Xue, Constance L. Cepko

**Affiliations:** Department of Genetics, Blavatnik Institute, and Department of Ophthalmology, Harvard Medical School, Boston, Massachusetts, USA. Howard Hughes Medical Institute, Chevy Chase, Maryland, USA.

**Keywords:** Ophthalmology, Therapeutics, Gene therapy, Neurodegeneration

## Abstract

Inherited retinal diseases, such as retinitis pigmentosa (RP), can be caused by thousands of different mutations, a small number of which have been successfully treated with gene replacement. However, this approach has yet to scale and may not be feasible in many cases, highlighting the need for interventions that could benefit more patients. Here, we found that microglial phagocytosis is upregulated during cone degeneration in RP, suggesting that expression of “don’t-eat-me” signals such as CD47 might confer protection to cones. To test this, we delivered an adeno-associated viral (AAV) vector expressing CD47 on cones, which promoted cone survival in 3 mouse models of RP and preserved visual function. Cone rescue with CD47 required a known interacting protein, signal regulatory protein α (SIRPα), but not an alternative interacting protein, thrombospondin-1 (TSP1). Despite the correlation between increased microglial phagocytosis and cone death, microglia were dispensable for the prosurvival activity of CD47, suggesting that CD47 interacts with SIRPα on nonmicroglial cells to alleviate degeneration. These findings establish augmentation of CD47/SIRPα signaling as a potential treatment strategy for RP and possibly other forms of neurodegeneration.

## Introduction

A major obstacle in developing treatments for inherited retinal diseases (IRDs) is the enormous genetic heterogeneity of pathogenic mutations. In the largest family of these disorders, retinitis pigmentosa (RP), thousands of unique mutations have been identified spanning approximately 100 different genes (RetNet; https://sph.uth.edu/retnet/). For a minority of IRDs, gene replacement therapy with adeno-associated viral (AAV) vectors has led to promising results in clinical trials and, in one instance, a successful commercial drug (1, 2). Nonetheless, this strategy has yet to reach the vast majority of patients and may not be feasible for those with less common, gain-of-function, or unidentified mutations.

Given these challenges, another approach to treating RP and other IRDs would be to develop mutation-agnostic therapies that preserve cone photoreceptors. In RP, disease mutations, many of which are exclusively expressed in rods, lead to the early death of these cells, often before the condition is diagnosed (3). Rod death is in itself tolerable, as rods are only needed for vision in dim light. However, rod loss is followed by the degeneration of cones, resulting in deterioration of daylight, color, and high-acuity vision. The reasons for secondary cone degeneration in RP remain incompletely understood but likely include increased oxidative stress (4, 5), decreased glucose availability (6–8), and immune dysregulation (9, 10). Regardless of the exact etiology, an intervention capable of protecting cones in RP independent of the initial genetic lesion would potentially be beneficial for many patients.

One of the hallmarks of neurodegenerative disorders, including RP, is the activation of microglia, the resident immune cells of the retina and central nervous system (11, 12). This activation upregulates phagocytosis and inflammatory cytokine production, facilitating the clearance of infections and cell debris, but also can damage nearby healthy tissue. In mice, activated microglia during the early stages of RP can engulf living rods, thereby hastening rod death (13, 14). While microglia are similarly activated during cone degeneration (9, 10), their effect on cone survival appears to be more nuanced. Previously, we showed that the antiinflammatory cytokine TGF-β1 requires microglia to preserve cones in RP mouse models (10), indicating that microglia, when properly induced, are capable of conferring protection to cones. However, we also found that depletion of microglia in untreated RP retinas had little effect on cone survival (9, 10), suggesting that if there are beneficial activities of microglia on cones, they are likely counterbalanced by detrimental ones. These findings left open the role of microglia in cone death and led us to ask whether microglial phagocytosis might contribute to cone demise.

Here, we tested if expression of an antiphagocytic molecule on cones could slow the rate of cone degeneration. Specifically, we used an AAV vector encoding CD47, a “don’t-eat-me” signal known to inhibit engulfment via interaction with signal regulatory protein α (SIRPα) on phagocytic cells (15, 16). Expression of CD47 preserved cones and vision in multiple mouse models of RP through a mechanism dependent on SIRPα but surprisingly not on microglia. Our findings support augmentation of CD47/SIRPα signaling as a possible mutation-agnostic therapy for RP and potentially other neurodegenerative diseases.

## Results

### Microglia show increased phagocytic activity during secondary cone degeneration.

**Figure 1:**
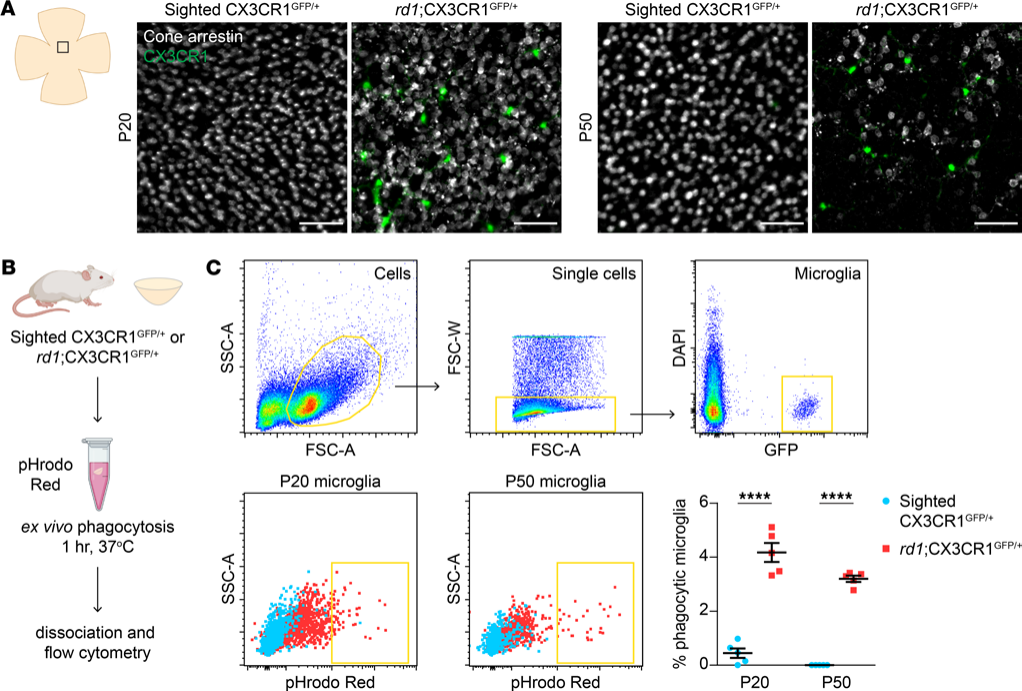
Cone degeneration is associated with increased microglial phagocytosis. (**A**) Cone arrestin immunostaining and CX3CR1-positive microglia in flat-mounted retinas from sighted CX3CR1^GFP/+^ (*rd1* heterozygous) and *rd1*;CX3CR1^GFP/+^ mice at P20 and P50. Images were acquired from the central retina at the level of the outer nuclear layer where cones reside. Scale bars: 50 μm. (**B**) Schematic of ex vivo phagocytosis assay. Retinas from sighted CX3CR1^GFP/+^ and *rd1*;CX3CR1^GFP/+^ mice were incubated with yeast (zymosan) particles conjugated to pHrodo Red, a pH-sensitive dye that fluoresces upon lysosomal acidification. Microglia were subsequently analyzed by flow cytometry. (**C**) Flow cytometry gating for microglia and quantification of microglial phagocytosis in sighted CX3CR1^GFP/+^ (*n* = 5) and *rd1*;CX3CR1^GFP/+^ (*n* = 5) retinas at P20 and P50. Data are shown as mean ± SEM. ****P < 0.0001 by 2-tailed Student’s *t* test.

We previously generated *rd1*;CX3CR1^GFP/+^ and sighted CX3CR1^GFP/+^ (*rd1* heterozygous) mice by crossing the widely used *rd1* model of RP with CX3CR1^GFP^ animals, in which microglia express GFP (9, 17, 18). To assess if microglia during the later stages of RP might phagocytose cones, retinas from these mice were immunostained for cone arrestin, a marker of all cones. In sighted CX3CR1^GFP/+^ retinas, microglia lacked any appreciable contact with cone arrestin–positive cells (Figure 1A), consistent with the normal exclusion of microglia from the photoreceptor layer (19). In contrast, microglia in *rd1*;CX3CR1^GFP/+^ retinas were often directly adjacent to degenerating cones, although in no case was overt engulfment of cones observed. To more sensitively measure microglial phagocytic activity, we explanted retinas from *rd1*;CX3CR1^GFP/+^ and sighted CX3CR1^GFP/+^ animals and incubated them with yeast particles conjugated to pHrodo Red, a pH-dependent dye that fluoresces upon lysosomal acidification (Figure 1B and ref. 20). Microglia from these retinas were then analyzed by flow cytometry at P20, the approximate age at which cone death in *rd1* mice begins (6), as well as P50, after substantial cone loss has occurred. At both time points, microglia from *rd1*;CX3CR1^GFP/+^ retinas internalized significantly more yeast than those from sighted controls (Figure 1C). Microglia thus exhibit increased phagocytic activity during secondary cone degeneration, a factor we hypothesized might worsen cone demise.

### Expression of the CD47 don’t-eat-me signal promotes survival of degenerating cones.

**Figure 2:**
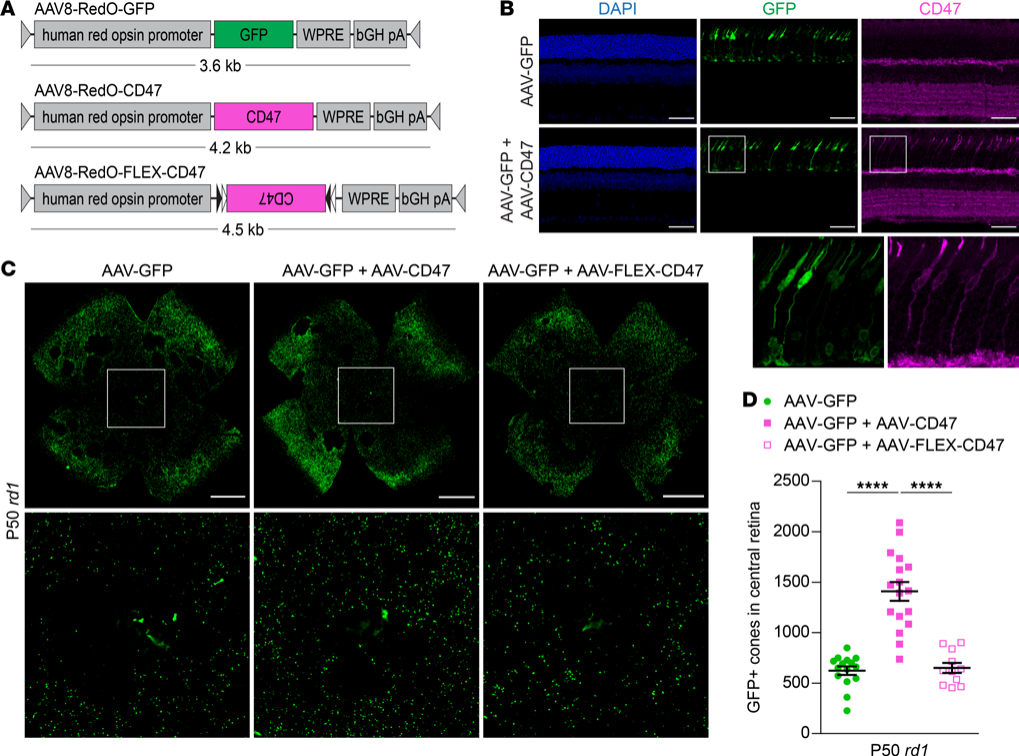
Effect of CD47 expression on cone survival. (**A**) Schematics of AAV vectors. AAV8-RedO-FLEX-CD47 is a flip-excision (FLEX) vector in which the CD47 transgene is inverted and flanked by lox2272 (black triangles) and loxP (white triangles) sites. (**B**) Immunostaining for CD47 in P40 wild-type (CD-1) retinas following infection with AAV8-RedO-GFP or AAV8-RedO-GFP plus AAV8-RedO-CD47. Nuclei were labeled with DAPI. Scale bars: 50 μm. (**C**) Representative flat mounts of P50 *rd1* retinas following infection with AAV8-RedO-GFP, AAV8-RedO-GFP plus AAV8-RedO-CD47, or AAV8-RedO-GFP plus AAV8-RedO-FLEX-CD47. Paired images depict low- and high-magnification views. Scale bars: 1 mm. (**D**) Quantification of GFP-positive cones in central retinas of *rd1* mice (*n* = 11–17) following infection with AAV8-RedO-GFP, AAV8-RedO-GFP plus AAV8-RedO-CD47, or AAV8-RedO-GFP plus AAV8-FLEX-RedO-CD47. Data are shown as mean ± SEM. ****P < 0.0001 by 2-tailed Student’s *t* test.

Among the key regulators of phagocytosis are don’t-eat-me signals, such as CD47, which when present on cells impede their engulfment by macrophages (21). To test if inhibiting phagocytosis during RP might benefit cones, we created an AAV vector (AAV8-RedO-CD47) using the human red opsin promoter to express CD47 on cones (Figure 2A). In wild-type mice injected subretinally with a GFP control vector (AAV8-RedO-GFP), which labels both M- and S-type cones (8), endogenous CD47 could be seen in retinal plexiform layers, as previously described, but not in photoreceptors (Figure 2B and ref. 22). Following coadministration of AAV8-RedO-GFP and AAV8-RedO-CD47, CD47 immunostaining could additionally be detected in cones. Notably, use of AAV8-RedO-CD47 in wild-type mice produced no obvious changes in cone, retinal, or retinal pigment epithelium (RPE) morphology when evaluated more than 3 months later (Supplemental Figure 1; supplemental material available online with this article; https://doi.org/10.1172/jci.insight.150796DS1).

In mouse models of RP, cone degeneration proceeds from the optic nerve head outward with relative sparing of the peripheral retina. To measure the effect of CD47 on cone survival, GFP-positive cones in the central retinas of *rd1* mice were quantified. Cone counting using a GFP vector has been previously validated by our group and provides unambiguous labeling of cones, enabling automated quantification (4, 9, 10, 23). At the doses used in this study, coinfection with 2 serotype 8 vectors in cones is expected to be at least 90% (4). Compared with infection with AAV8-RedO-GFP alone, coinfection with AAV8-RedO-CD47 approximately doubled the number of cones in the central retina at P50 (Figure 2, C and D). In contrast, coinfection with AAV8-RedO-FLEX-CD47, a control vector with the CD47 sequence inverted, did not significantly change the number of remaining cones. To assess cone preservation with CD47 beyond the central retina, entire *rd1* retinas were next analyzed for GFP-positive cones using flow cytometry (Supplemental Figure 2). Consistent with the histological findings, this method showed greater cone counts at P50 with AAV8-RedO-GFP plus AAV8-RedO-CD47 than AAV8-RedO-GFP only (Supplemental Figure 2). To also gauge cone survival versus retinas from untreated eyes, *rd1* retinas at P50 were immunostained for cone arrestin. Relative to central retinas without treatment or receiving AAV8-RedO-GFP alone, addition of AAV8-RedO-CD47 again resulted in more cones, as defined by this marker (Supplemental Figure 3). Together, these data demonstrated that CD47 expression could promote survival of cones in *rd1* mice.

### CD47 promotes cone survival and retention of vision in multiple genetic models.

**Figure 3:**
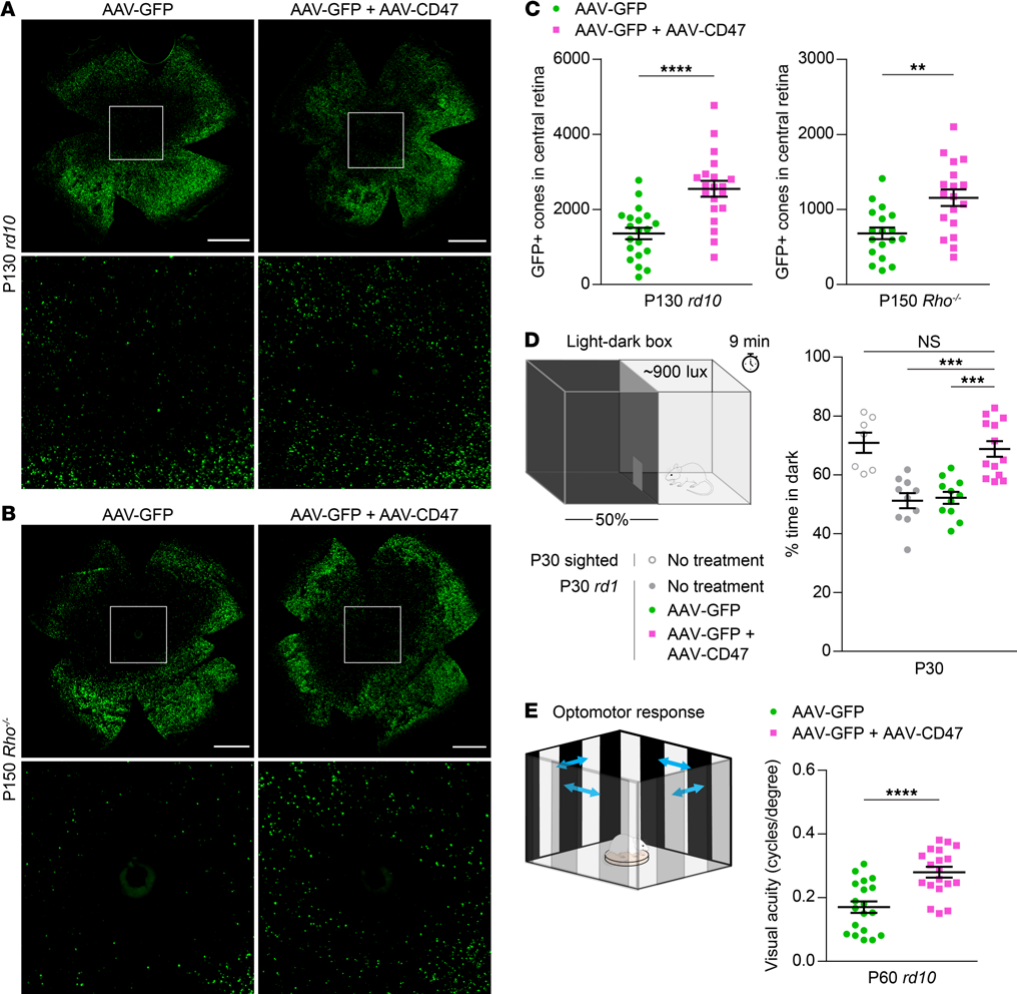
Effect of CD47 expression on long-term cone survival and visual function. (**A**) Representative flat mounts of P130 *rd10* retinas following infection with AAV8-RedO-GFP or AAV8-RedO-GFP plus AAV8-RedO-CD47. Paired images depict low- and high-magnification views. Scale bars: 1 mm. (**B**) Representative flat mounts of P150 *Rho^–/–^* retinas following infection with AAV8-RedO-GFP or AAV8-RedO-GFP plus AAV8-RedO-CD47. Paired images depict low- and high-magnification views. Scale bars: 1 mm. (**C**) Quantification of GFP-positive cones in central retinas of *rd10* (*n* = 20) and *Rho^–/–^* (*n* = 18) mice following infection with AAV8-RedO-GFP or AAV8-RedO-GFP plus AAV8-RedO-CD47. (**D**) Percentage time spent in dark in a 50:50 light-dark box for untreated (*n* = 7–10) and *rd1* (*n* = 11–13) mice following infection with AAV8-RedO-GFP or AAV8-RedO-GFP plus AAV8-RedO-CD47. (**E**) Visual thresholds in eyes from P60 *rd10* mice (*n* = 19), as measured by optomotor following infection with AAV8-RedO-GFP or AAV8-RedO-GFP plus AAV8-RedO-CD47. Data are shown as mean ± SEM. **P < 0.01, ***P < 0.001, ****P < 0.0001 by (**C** and **E**) 2-tailed Student’s *t* test and (**D**) 2-tailed Student’s *t* test with Bonferroni correction.

To determine whether CD47 might similarly slow retinal degeneration in other models of RP, AAV8-RedO-CD47 was tested in *rd10* mice, which carry a missense mutation in *Pde6b*, and *Rho^–/–^* mice, which lack rhodopsin. In both *rd10* retinas at P100 and P130 and *Rho^–/–^* retinas at P150, AAV8-RedO-CD47 again improved the number of cones (Figure 3, A–C, and Supplemental Figure 2), suggesting that CD47 may generically combat cone degeneration. The effect of AAV8-RedO-CD47 on rods was also examined in *rd10* animals by measuring the thickness of the outer nuclear layer, which reflects the number of remaining rods. Cone expression of CD47 did not appreciably slow rod death in these mice (Supplemental Figure 4), indicating that prolonged cone survival with AAV8-RedO-CD47 was not due to an increase in the number of rods.

To investigate the therapeutic relevance of AAV8-RedO-CD47, treated animals were then subjected to 2 vision-dependent behavioral assays. First, a light-dark discrimination test was performed by leveraging the natural preference of sighted mice for dark rather than well-illuminated spaces (see Methods). In keeping with this, wild-type animals spent approximately 70% of their time in the dark half of a 50:50 light-dark environment, while *rd1* mice without treatment or receiving only AAV8-RedO-GFP divided their time evenly between the 2 chambers (Figure 3D). Compared with these latter 2 groups, *rd1* animals receiving AAV8-RedO-GFP plus AAV8-RedO-CD47 spent significantly more time in the dark, consistent with better preservation of visual function. As a second measure of vision, mice were evaluated using an optomotor assay in which moving stripes were presented to elicit the vision-dependent optomotor response. By varying the spatial frequency of stripes to make them easier or more difficult to see, the visual threshold in each eye could be estimated (24). In *rd10* animals tested at P60, visual thresholds were significantly higher in eyes infected with AAV8-RedO-GFP plus AAV8-RedO-CD47 compared with those infected with AAV8-RedO-GFP alone (Figure 3E), again suggesting better retention of sight. CD47 expression thus not only promotes cone survival in different mouse models of RP, but also protects from vision loss, supporting its potential use as a mutation-agnostic therapy for this condition.

### Delayed expression of CD47 ameliorates cone death.

**Figure 4:**
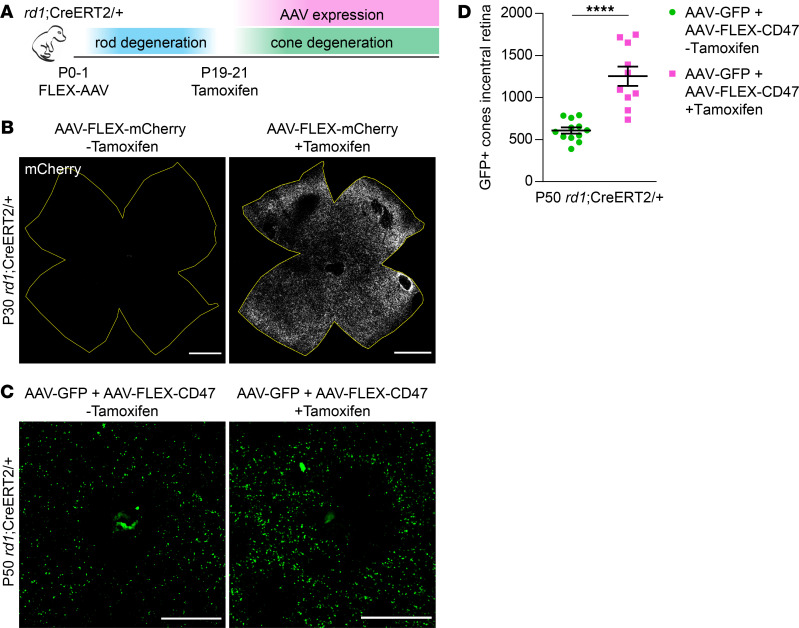
Effect of delayed CD47 expression on cone survival. (**A**) Schematic of delayed AAV expression experiments. P0–P1 *rd1*;CreERT2/+ mice were subretinally injected with FLEX vectors, which were subsequently activated by i.p. injections of tamoxifen from P19–P21. (**B**) Representative flat mounts of P30 *rd1*;CreERT2/+ retinas following infection with AAV8-RedO-FLEX-mCherry with or without i.p. injections of tamoxifen. Boundaries of each retina are depicted in yellow. Scale bars: 1 mm. (**C**) Representative images of central retinas from P50 *rd1*;CreERT2/+ mice following infection with AAV8-RedO-GFP plus AAV8-RedO-FLEX-CD47 with or without i.p. injections of tamoxifen. Scale bars: 500 μm. (**D**) Quantification of GFP-positive cones in central retinas of *rd1*;CreERT2/+ mice (*n* = 10–12) following infection with AAV8-RedO-GFP plus AAV8-RedO-FLEX-CD47 with or without i.p. injections of tamoxifen. Data are shown as mean ± SEM. ****P < 0.0001 by 2-tailed Student’s *t* test.

Clinically, patients with RP are often diagnosed following the development of night blindness (3). We therefore asked whether CD47 could still protect cones even after the majority of rods have died. To model this scenario, we bred R26-CreERT2 mice, which undergo inducible Cre activation in the presence of tamoxifen, with the *rd1* strain to obtain *rd1*;CreERT2/+ animals. When combined with a flip-excision (FLEX) vector (25), this approach allowed for delayed AAV expression while avoiding the technical challenges of subretinal injections in older mice. As a test for CreERT2-mediated recombination, P0–P1 *rd1*;CreERT2/+ animals were infected with AAV8-RedO-FLEX-mCherry, a vector designed to express mCherry only after exposure to tamoxifen (Figure 4A). In the absence of tamoxifen, AAV8-RedO-FLEX-mCherry produced no detectable fluorescence after 30 days (Figure 4B). However, the same vector with tamoxifen from P19–P21 led to robust mCherry expression throughout the retina. Using this strategy and an analogous AAV8-RedO-FLEX-CD47 vector, the effect of delayed CD47 expression on cone survival was examined. In *rd1*;CreERT2/+ animals coinfected with AAV8-RedO-GFP plus AAV8-RedO-FLEX-CD47 but without tamoxifen, the number of cones at P50 was comparable to that seen in *rd1* mice (Figure 2D and Figure 4, C and D). In contrast, in animals additionally receiving tamoxifen from P19–P21, an age by which most rods have died (6), significantly more GFP-positive cones were observed. Collectively, these findings suggest that CD47 may alleviate cone degeneration even if administered later in the disease progression.

**Figure 5:**
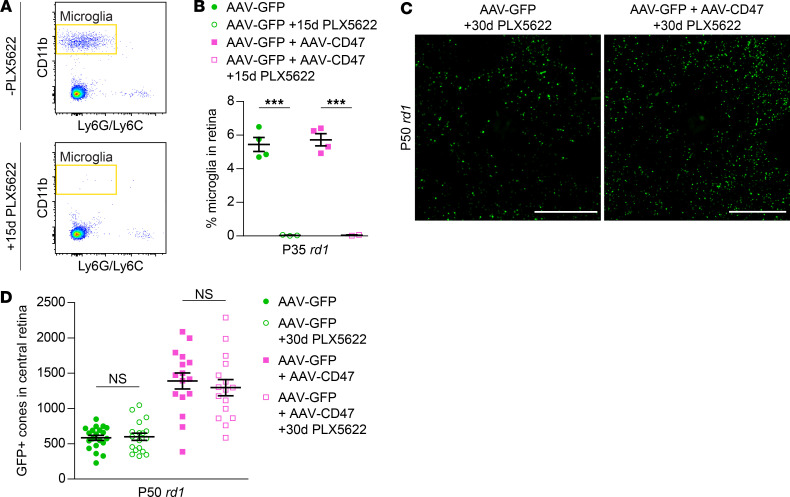
Role of microglia in CD47-mediated cone survival. (**A**) Flow cytometry gating for retinal microglia from P35 *rd1* mice with or without PLX5622 from P20–P34. Microglia were defined as CD11b-positive, Ly6G/Ly6C-negative cells. (**B**) Quantification by flow cytometry of retinal microglia from P35 *rd1* mice (*n* = 2–4) following infection with AAV8-RedO-GFP or AAV8-RedO-GFP plus AAV8-RedO-CD47 with or without PLX5622 from P20–P34. (**C**) Representative images of central retinas from P50 *rd1* mice following infection with AAV8-RedO-GFP or AAV8-RedO-GFP plus AAV8-RedO-CD47 and PLX5622 from P20–P49. Scale bars: 500 μm. (**D**) Quantification of GFP-positive cones in central retinas of *rd1* mice (*n* = 16–18) following infection with AAV8-RedO-GFP or AAV8-RedO-GFP plus AAV8-RedO-CD47 and PLX5622 from P20–P49. Data from groups of mice without PLX5622 infection were taken from Figure 2D. Data are shown as mean ± SEM. ***P < 0.001 by 2-tailed Student’s *t* test.

*CD47 requires SIRPαbut not microglia or TSP1 to preserve cones*. In the brain, the don’t-eat-me function of CD47 is thought to be largely mediated by microglia (26, 27). We thus suspected that AAV8-RedO-CD47 might protect cones by inhibiting their phagocytosis by microglia. To investigate this mechanism, mice infected subretinally with AAV8-RedO-CD47 had their microglia pharmacologically depleted using PLX5622, a small molecule blocking a receptor on microglia essential for their survival (28). In the retina, PLX5622 depleted approximately 99% of microglia within 15 days (Figure 5, A and B), demonstrating virtually complete elimination of this cell type. Consistent with our previous observations (10), PLX5622 administration in *rd1* mice from P20 to P49 had no significant effect on cone counts in eyes receiving only AAV8-RedO-GFP (Figure 5, C and D). Surprisingly, PLX5622 also failed to perturb cone preservation with AAV8-RedO-CD47, indicating that microglia were dispensable for its mechanism of action.

**Figure 6:**
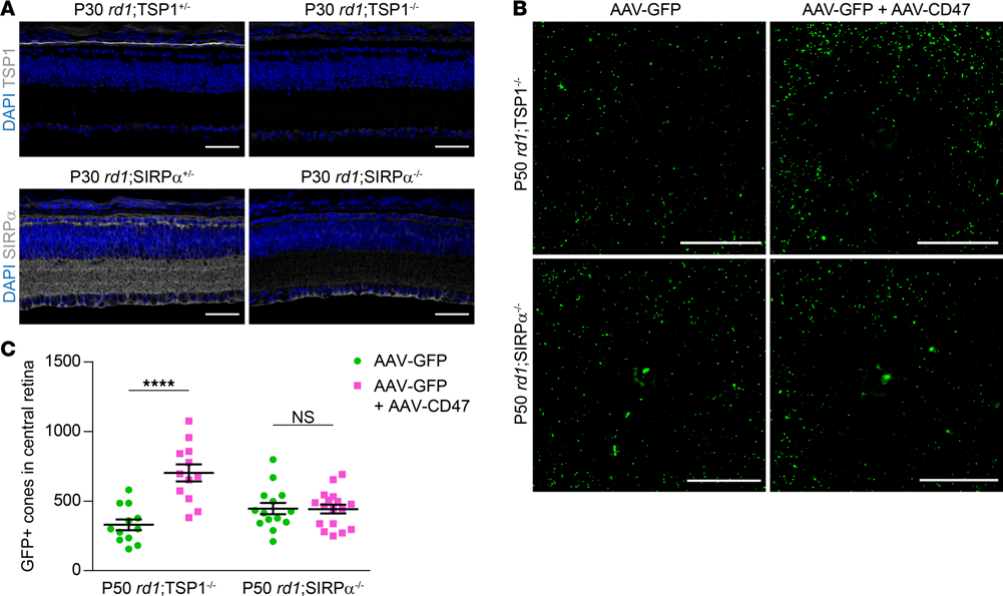
Role of TSP1 and SIRPα in CD47-mediated cone survival. (**A**) Immunostaining for TSP1 and SIRPα, respectively, in P30 *rd1*;TSP1^–/–^ and *rd1*;SIRPα^–/–^ retinas or heterozygous controls. Scale bars: 50 μm. (**B**) Representative images of central retinas from P50 *rd1*;TSP1^–/–^ and *rd1*;SIRPα^–/–^ mice following infection with AAV8-RedO-GFP or AAV8-RedO-GFP plus AAV8-RedO-CD47. Scale bars: 500 μm. (**C**) Quantification of GFP-positive cones in central retinas of *rd1*;TSP1^–/–^ (*n* = 12) and *rd1*;SIRPα^–/–^ (*n* = 14–17) mice following infection with AAV8-RedO-GFP or AAV8-RedO-GFP plus AAV8-RedO-CD47. Data are shown as mean ± SEM. ****P < 0.0001 by 2-tailed Student’s *t* test.

There are 2 major pathways by which CD47 is known to mediate intercellular signaling. Through its extracellular domain, CD47 can interact with SIRPα, a transmembrane protein expressed on a broad range of cell types, including microglia, macrophages, dendritic cells, and neurons (29). In addition, CD47 can be activated by thrombospondin-1 (TSP1), a secreted protein; the binding of TSP1 to CD47 has been shown to help resolve subretinal inflammation (30). To determine if either TSP1/CD47 or CD47/SIRPα signaling were necessary for CD47 to save cones, we generated *rd1*;TSP1^–/–^ and *rd1*;SIRPα^–/–^ mice, which exhibited loss of retinal TSP1 and SIRPα, respectively (Figure 6A). In *rd1*;TSP1^–/–^ animals, coinfection with AAV8-RedO-GFP plus AAV8-RedO-CD47 doubled the number of remaining cones relative to that in animals infected with AAV8-RedO-GFP alone (Figure 6, B and C). In contrast, in *rd1*;SIRPα^–/–^ mice, no difference in cone survival was observed with or without AAV8-RedO-CD47. These results demonstrate that the therapeutic effect of AAV8-RedO-CD47 requires SIRPα but not TSP1. Protection of cones with CD47 therefore likely occurs via increased CD47/SIRPα signaling involving one or more nonmicroglial cell types.

## Discussion

Despite a growing number of gene therapy programs targeting IRDs, the vast majority of patients still lack effective treatment. Correcting individual genes or mutations may improve this situation, but only for subgroups who have those specific lesions, highlighting the need for an intervention that can benefit many more people. In this study, we developed AAV8-RedO-CD47, a gene therapy vector that protected cones and vision in multiple models of RP (Supplemental Table 1), suggesting it as a potential generic treatment for patients with different mutations. Importantly, delayed expression of CD47 until after most rods had died also preserved cones, implying that this therapy would still be beneficial if administered later in the disease progression. AAV8-RedO-CD47 may thus offer a possible treatment option for many patients with RP, including those who are diagnosed at older ages or with genetics that preclude straightforward gene replacement or repair. The vector might further help combat cone death in other IRDs and degenerative retinal disorders, such as age-related macular degeneration, which affects an estimated 200 million people worldwide (31).

One key question is the mechanism by which CD47 makes cones more resistant to degeneration. While we initially hypothesized that better cone survival would be secondary to reduced engulfment by microglia, this explanation is incompatible with the PLX5622 experiments, in which near-complete elimination of retinal microglia failed to perturb CD47-mediated protection of cones. Additionally, although cones may have been exposed to TSP1 secreted from the adjacent RPE, AAV8-RedO-CD47 still preserved cones in the absence of TSP1/CD47 signaling. Instead, cone rescue with AAV8-RedO-CD47 required SIRPα, indicating that CD47 on cones likely interacts with SIRPα on nonmicroglial cells to alleviate degeneration. Outside of microglia, SIRPα in the eye is normally expressed in the synapse-rich plexiform layers of the retina (22). However, the protein is also present on multiple immune cell populations, including monocytes, macrophages, neutrophils, and dendritic cells, and has more recently been described on natural killer cells and a subset of cytotoxic T cells (29, 32, 33). Binding of CD47 to SIRPα on immune cell populations consistently acts as a negative checkpoint, whether by inhibiting phagocytosis, preventing cell killing, or impeding recruitment of adaptive immunity (29). While Müller glia, another cell type in the retina, have also been shown to phagocytose photoreceptors (34), these cells do not appreciably express SIRPα (35). As a preliminary experiment, we used quantitative PCR to see if any SIRPα-positive immune subsets were enriched in the degenerating retina. These data showed upregulation of markers associated with dendritic cells (*Itgax*) and cytotoxic T cells (*Cd8a*; Supplemental Figure 5 and Supplemental Table 2). We therefore suspect that increased CD47/SIRPα signaling with AAV8-RedO-CD47 may save cones by suppressing dysregulated immune cells that would otherwise contribute to cone demise.

Where does CD47 fit in the landscape of emerging treatments for retinal degeneration? In addition to immune dysregulation, cones in RP appear to face a shortage of glucose, as evidenced by greater cone survival with interventions that boost glucose uptake or the use of alternative fuels (6–8). Degenerating cones further experience increased oxidative stress after the death of rods and may be helped by genes or small molecules with antioxidant activity (4, 5, 36). It is possible that combining CD47/SIRPα augmentation with some of these approaches could be additive or synergistic for saving cones. Moreover, technologies improving the tolerability and immunogenicity of AAV vectors might enable patients to benefit from sequential treatments (37, 38), such as AAV8-RedO-CD47 upon diagnosis of disease and optogenetic gene therapy at a later date (39).

Finally, therapeutic interest in the CD47/SIRPα axis has grown tremendously in recent years following the discovery that many cancers co-opt CD47 expression to evade antitumor immunity (40). Blocking CD47 and SIRPα have since become promising avenues to induce killing of tumor cells (41, 42), with several modalities now being trialed in patients (43, 44). Rather than disrupt, here we augmented CD47/SIRPα signaling in the eye by using an AAV vector to express CD47. This strategy was able to slow progression in multiple models of a blinding disease and may likewise be applicable to other neurodegenerative conditions. For example, CD47 levels have been found to be low in active lesions in multiple sclerosis (45), hinting that increasing CD47 in these regions might ameliorate neuroinflammation. It would similarly be interesting to see if CD47 expression could aid other cell types that undergo degeneration, such as retinal ganglion cells in glaucoma or motor neurons in amyotrophic lateral sclerosis.

## Methods

Further information can be found in Supplemental Methods.

### Mice.

CD-1 (strain 022) and FVB (strain 207) mice were purchased from Charles River Laboratories. CX3CR1^GFP^ (stock no. 005582), *rd10* (stock no. 004297), C3H (stock no. 000659), sighted C3H (stock no. 003648), R26-CreERT2 (stock no. 008463), TSP1^–/–^ (stock no. 006141), and sighted FVB (stock no. 004828) mice were purchased from The Jackson Laboratory. *Rho^–/–^* mice were a gift from Janis Lem (Tufts University, Boston, Massachusetts, USA) (46). SIRPα^–/–^ mice were a gift from Beth Stevens (Harvard Medical School) (26). CX3CR1^GFP^, R26-CreERT2, TSP1^–/–^, and SIRPα^–/–^ lines were crossed with FVB mice for at least 4 generations to obtain the following strains: sighted CX3CR1^GFP/+^, *rd1*;CX3CR1^GFP/+^, *rd1*;CreERT2/+, *rd1*;TSP1^+/-^, *rd1*;TSP1^–/–^, *rd1*;SIRPα^+/-^, and *rd1*;SIRPα^–/–^. Genotyping was performed by Transnetyx using real-time PCR. Mice were maintained at Harvard Medical School on a 12-hour alternating light and dark cycle. Animals were housed in standard ventilated racks at a density of up to 5 per cage. Both male and female animals were used in all experiments and were randomly assigned to experimental groups.

### Histology.

Retinal flat mounts from *rd1* (FVB), *rd10*, and *Rho^–/–^* mice were prepared as previously described (9, 10). Following dissection of enucleated eyes in PBS, retinas were fixed in 4% paraformaldehyde for 30 minutes at room temperature, washed twice with PBS, and relaxed with 4 radial incisions. For cone arrestin immunostaining, retinas were additionally blocked with 5% donkey serum and 0.3% Triton X-100 in PBS for 1 hour at room temperature, incubated with 1:3000 of anti–cone arrestin (Millipore, AB15282) in blocking solution overnight at 4°C, and labeled with 1:1000 of donkey anti-rabbit secondary (Jackson ImmunoResearch, 711-585-152) in PBS for 2 hours at room temperature. Retinas were mounted on microscope slides using Fluoromount-G (SouthernBiotech) with the ganglion cell layer facing up. For retinal cross-sections, enucleated eyes were dissected to remove the cornea, iris, lens, and ciliary body. The remaining eye cups were cryoprotected in a sucrose gradient, frozen in a 1:1 mixture of optimal cutting temperature compound (Tissue-Tek) and 30% sucrose in PBS, and sectioned on a Leica CM3050S cryostat (Leica Microsystems) at a thickness of 20 μm. Tissues were blocked with 5% donkey serum and 0.1% Triton X-100 in PBS for 1 hour at room temperature and incubated with 1:1000 of anti-CD47 (BD Biosciences, 555297), 1:500 of anti-TSP1 (Abcam, ab85762), or 1:500 of anti-SIRPα (QED Bioscience, 2428) in blocking solution overnight at 4°C. Sections were subsequently labeled with 1:1000 of the appropriate secondary antibody, [Alexa Fluor 594 AffiniPure Donkey Anti-Rabbit IgG (H+L) and Alexa Fluor 594 AffiniPure Donkey Anti-Rat IgG (H+L), Jackson ImmunoResearch, 711-585-152 or 712-585-153] in PBS for 2 hours at room temperature, followed by 0.5 μg/mL DAPI (Thermo Fisher Scientific) in PBS for 5 minutes at room temperature before mounting.

### Ex vivo phagocytosis assay.

Freshly dissected retinas were incubated with gentle agitation for 1 hour at 37°C in 1 mg/mL pHrodo Red–conjugated zymosan bioparticles (Thermo Fisher Scientific) resuspended in retinal culture media (1:1 mixture of DMEM and F-12 supplemented with L-glutamine, B27, N2, and penicillin-streptomycin). Retinas were subsequently washed with PBS and dissociated using cysteine-activated papain as previously described (9). After 2 additional washes, samples were passed through a 40 μm filter and stained with 0.5 μg/mL DAPI in FACS buffer (2 mM EDTA and 2% fetal bovine serum in PBS) to exclude nonviable cells. Data were collected on a Cytek DxP11 cytometer and analyzed using FlowJo 10. CX3CR1-positive microglia were defined as phagocytic if positive for pHrodo Red.

### AAV vector cloning and production.

The AAV-human red opsin-GFP-WPRE-bGH (AAV8-RedO-GFP) plasmid, containing the promoter/enhancer element identified by Jeremy Nathans (Johns Hopkins University, Baltimore, Maryland, USA) and characterized by Li et al. (47), was a gift from Botond Roska (Institute of Molecular and Clinical Ophthalmology Basel, Basel, Switzerland; ref. 39). The AAV8-RedO-CD47 plasmid was cloned by replacing the GFP-coding sequence in AAV8-RedO-GFP with that of the most abundant isoform of mouse CD47 (NM_010581.3; ref. 48). The AAV8-RedO-FLEX-mCherry plasmid was cloned by replacing the GFP-coding sequence in AAV8-RedO-GFP with that of mCherry inverted and flanked by lox2272 and loxP sites. The AAV8-RedO-FLEX-CD47 plasmid was cloned by replacing the inverted mCherry sequence in AAV8-RedO-FLEX-mCherry with that of inverted CD47. AAV vectors were generated as previously described by transfecting 293T cells (ATCC) with a mixture the vector plasmid, adenovirus helper plasmid, and rep2/cap8 packaging plasmid (49, 50). Seventy-two hours after transfection, viral particles were harvested from the supernatant, PEGylated overnight, precipitated by centrifugation, treated with Benzonase nuclease (MilliporeSigma), and purified through an iodixanol gradient before collection in 100–200 μL PBS. Vectors were semiquantitatively titered by SYPRO Ruby (Molecular Probes) for viral capsid proteins (VP1, VP2, and VP3) relative to a reference vector titered by real-time PCR.

### AAV vector delivery.

Subretinal injections of AAV vectors were performed on neonatal mice (P0–P1) as previously described (51). Following anesthetization of the animal on ice, the palpebral fissure was gently opened with a 30-gauge needle and the eye was exposed. Using a glass needle controlled by a FemtoJet microinjector (Eppendorf), approximately 0.25 μL of vectors were then delivered into the subretinal space. Both left and right eyes were used for injections. AAV8-RedO-GFP was administered at approximately 5 × 10^8^ vector genomes per eye, a dose capable of transducing up to 99% of cones (4). All other vectors were administered at approximately 1 × 10^9^ vector genomes per eye.

### Image acquisition and analysis.

Retinal flat mounts were imaged using a Nikon Ti inverted wide-field microscope (×10 or ×20 air objective). Retinal cross-sections were imaged using a Zeiss LSM710 scanning confocal microscope (×20 air objective or ×40 oil objective). All image analysis was performed using ImageJ (NIH). To quantify GFP-positive or cone arrestin–positive cones, custom ImageJ modules were used as previously described (9, 10). For each flat mount, the locations of the optic nerve head and 4 retinal leaflets were first manually defined. The number of GFP-positive or cone arrestin–positive objects within the region corresponding to the central retina was then automatically counted and used to represent the number of cones in that sample.

### Light-dark test.

Light avoidance in *rd1* (C3H) and sighted C3H mice following no treatment or treatment in both eyes was assessed as previously described (10). Testing was not performed in *rd10* animals, as light avoidance in this strain is preserved for at least 4 months (data not shown). A plastic chamber (Med Associates) measuring 28 cm (length) by 28 cm (width) by 21 cm (height) was divided into 2 equally sized compartments: 1 dark and 1 brightly illuminated (~900 lux). The 2 compartments were connected by a small opening and differed in temperature by less than 1°C. At the start of each trial, a mouse was placed in the illuminated compartment and its activity recorded for 10 minutes. If after 1 minute, the animal had not yet entered the dark compartment, it was gently guided there and removed from the chamber, and the trial was restarted. Mice were tracked using infrared sensors and location data were analyzed with Activity Monitor (Med Associates). Percentage of time spent in dark was calculated based on the final 9 minutes of each trial.

### Optomotor assay.

Optomotor responses are absent in the *rd1* strain (52) and were consequently assessed in *rd10* mice. Visual thresholds were measured by an observer blinded to the treatment groups using the OptoMotry System (CerebralMechanics) as previously described (9). Animals were placed in a chamber with bright background luminance to saturate rods and presented with moving gratings of varying spatial frequencies. Left and right eyes were assessed using clockwise and counterclockwise gratings, respectively, as the optomotor response is evoked by temporal-to-nasal motion in mice (24). For each eye, the highest spatial frequency at which the mouse tracked the grating was determined to be the visual threshold.

### Tamoxifen injections.

Tamoxifen (MilliporeSigma) was dissolved in corn oil (MilliporeSigma) and dosed at 2 mg daily from P19–P21 via i.p. injections.

### Microglia depletion.

Microglia were depleted using PLX5622 (Plexxikon), an orally available CSF1R inhibitor. PLX5622 was formulated into AIN-76A rodent chow (Research Diets) at 1200 mg/kg and provided ad libitum during periods of depletion. For quantification of retinal microglia, freshly dissected retinas were dissociated using cysteine-activated papain as previously described (9). Cells were subsequently blocked with 1:100 anti-CD16/32 (BD Pharmingen, 553142) for 5 minutes on ice, followed by incubation with 1:200 PE-Cy5–conjugated anti-CD11b (BioLegend, 101209), 1:200 APC-Cy7–conjugated anti-Ly6C (BioLegend, 128025), and 1:200 APC-Cy7–conjugated anti-Ly6G (BioLegend, 127623) for 20 minutes on ice. After washes, samples were passed through a 40 μm filter and stained with 0.5 μg/mL DAPI in FACS buffer. Data were collected on a BD FACSAria II and analyzed using FlowJo 10.

### Statistics.

Statistical analyses were performed using GraphPad Prism software. Experimental groups were compared using 2-tailed Student’s *t* tests, with the addition of a Bonferroni correction if 3 or more comparisons were performed. A *P* value of less than 0.05 was considered statistically significant. Information on group data and replicates is reported in each figure legend.

### Study approval.

All animal experiments were conducted under protocols approved by the Institutional Animal Care and Use Committee of Harvard Medical School.

## Author contributions

SKW and CLC designed the study. SKW and YX performed the experiments and analyzed the data. SKW and CLC wrote the manuscript with input from YX.

## Supplementary Material

Supplemental data

## Version 1 07/01/2021

In-Press Preview

## Version 2 08/23/2021

Electronic publication
