# Supplementary material for: Augmentation of CD47/SIRPα signaling protects cones in genetic models of retinal degeneration
Source: JCI Insight. 2021 Aug 23;6(16):e150796. doi: 10.1172/jci.insight.150796 (PMC8409989; doi:10.1172/jci.insight.150796)
Supplement: Supplemental data [file jciinsight-6-150796-s048.pdf]

SUPPLEMENTAL METHODS

Retinal pigment epithelium (RPE) immunostaining. To assess the morphology of the RPE, enucleated eyes were dissected to remove the cornea, iris, lens, ciliary body, retina, and connective tissue. The remaining RPE-choroid-sclera complex was fixed in 4% paraformaldehyde for one hour at room temperature, blocked in PBS containing 5% donkey serum and 0.3% Triton X-100 for one hour, and stained with 1:100 of anti-ZO-1 (ThermoFisher Scientific, 61-7300) for two nights at 4°C. Samples were subsequently incubated with 1:1000 of donkey anti-rabbit secondary antibody (Jackson ImmunoResearch, 711-585-152) in PBS for two hours at room temperature, relaxed with four radial incisions, and flat-mounted onto microscope slides. Images of ZO-1 immunostaining in flat-mounted RPE preparations were acquired in the mid-periphery using a Zeiss LSM710 scanning confocal microscope (20x air objective) and displayed as maximum intensity projections.

Quantitative polymerase chain reaction (qPCR). RNA was isolated from *rd1* (FVB) and wild-type (sighted FVB) retinas using an RNeasy Micro Kit (Qiagen) with one whole retina collected per sample. cDNA was then synthesized using the SuperScript III First-Strand Synthesis System (Invitrogen) with oligo(dT) primers, and qPCR was performed using the Power SYBR Green PCR Master Mix (Applied Biosystems) on a CFX96 real-time PCR detection system (Bio-Rad). Reactions were conducted in triplicate with expression normalized to either *Gapdh*, a housekeeping gene, or *Thy1*, a marker for retinal ganglion cells. qPCR primer sequences are listed in Supplemental Table 2.

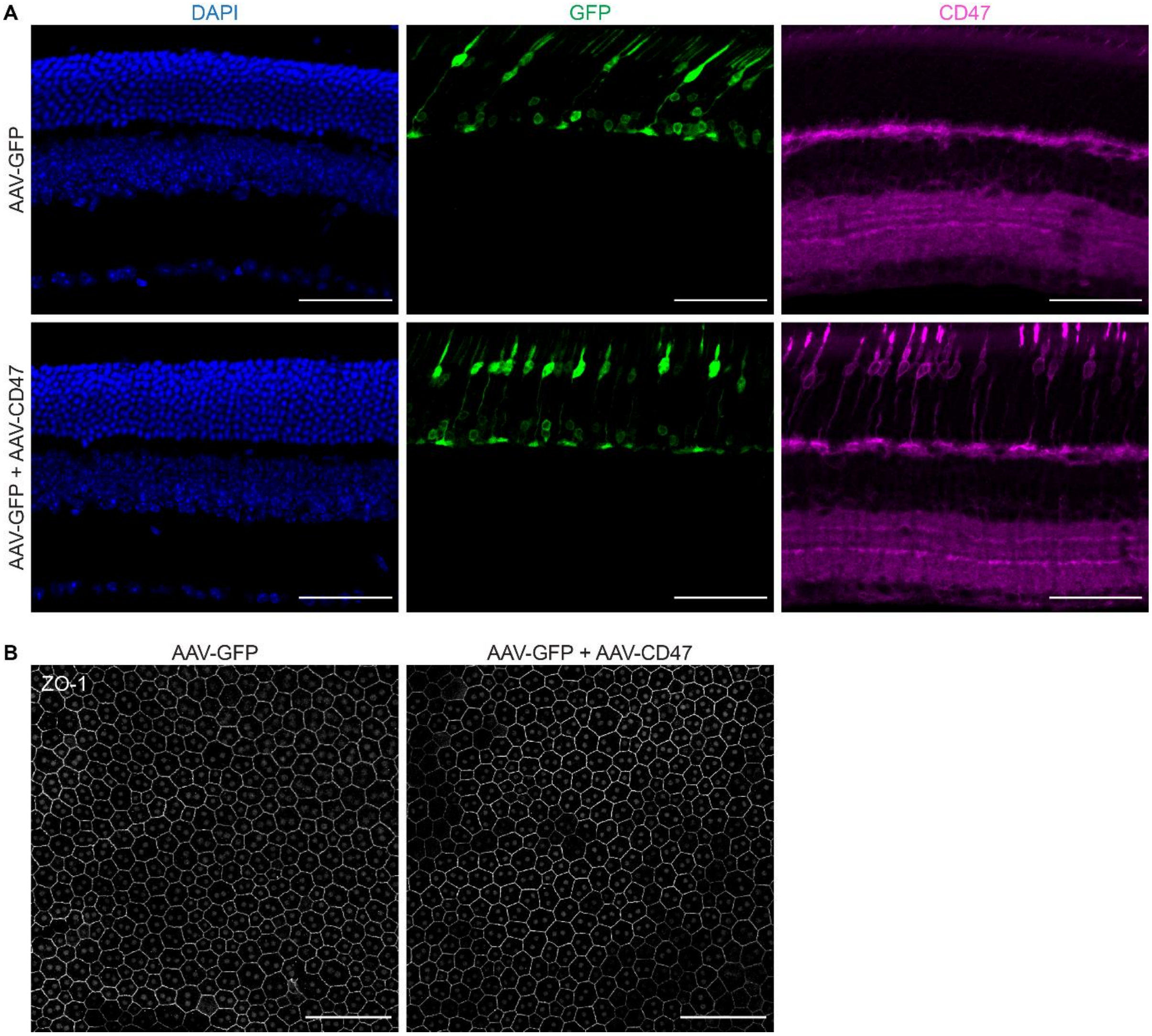

Supplemental Figure 1. Long-term expression of CD47 in wild-type retinas.

(**A**) Immunostaining for CD47 in P100 wild-type (CD-1) retinas following infection with AAV8-RedO-GFP or AAV8-RedO-GFP plus AAV8-RedO-CD47. Nuclei were labeled with 4′,6-diamidino-2-phenylindole (DAPI). Scale bars, 50 µm. (**B**) Immunostaining for ZO-1, a marker of epithelial tight junctions, in flat-mounted RPE preparations from P100 wild-type eyes following infection with AAV8-RedO-GFP or AAV8-RedO-GFP plus AAV8-RedO-CD47. Scale bars, 100 µm.

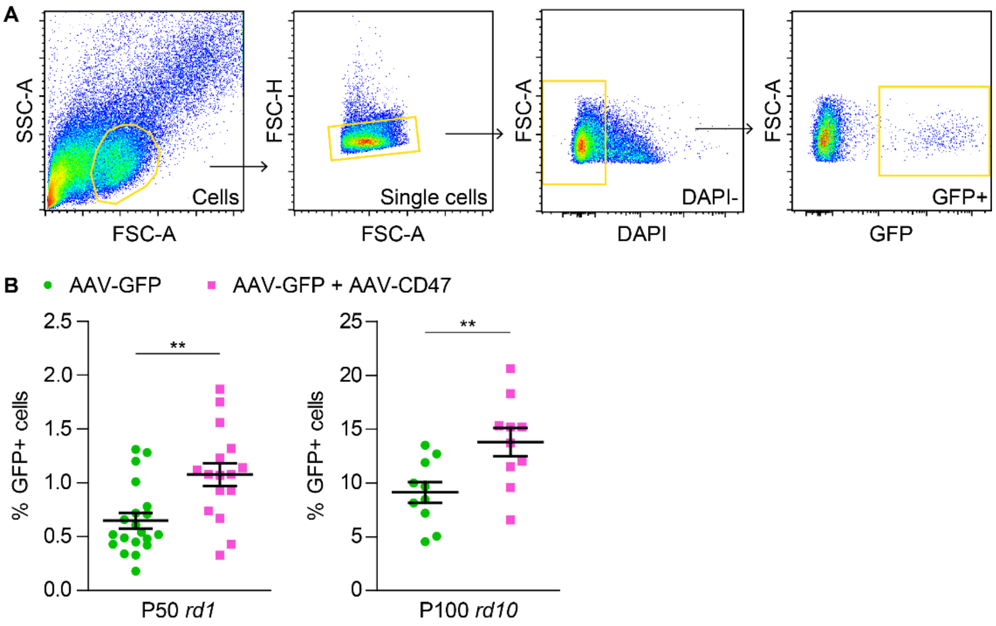

Supplemental Figure 2. Quantification of cone survival by flow cytometry.

(**A**) Flow cytometry gating for GFP-positive cones in *rd1* and *rd10* retinas following infection with AAV8-RedO-GFP or AAV8-RedO-GFP plus AAV8-RedO-CD47. (**B**) Quantification by flow cytometry of GFP-positive cones in P50 *rd1* (*n* = 16-20) and P100 *rd10* (*n* = 10) retinas following infection with AAV8-RedO-GFP or AAV8-RedO-GFP plus AAV8-RedO-CD47. Data are shown as mean ± SEM. ** *P*<0.01 by two-tailed Student's t-test.

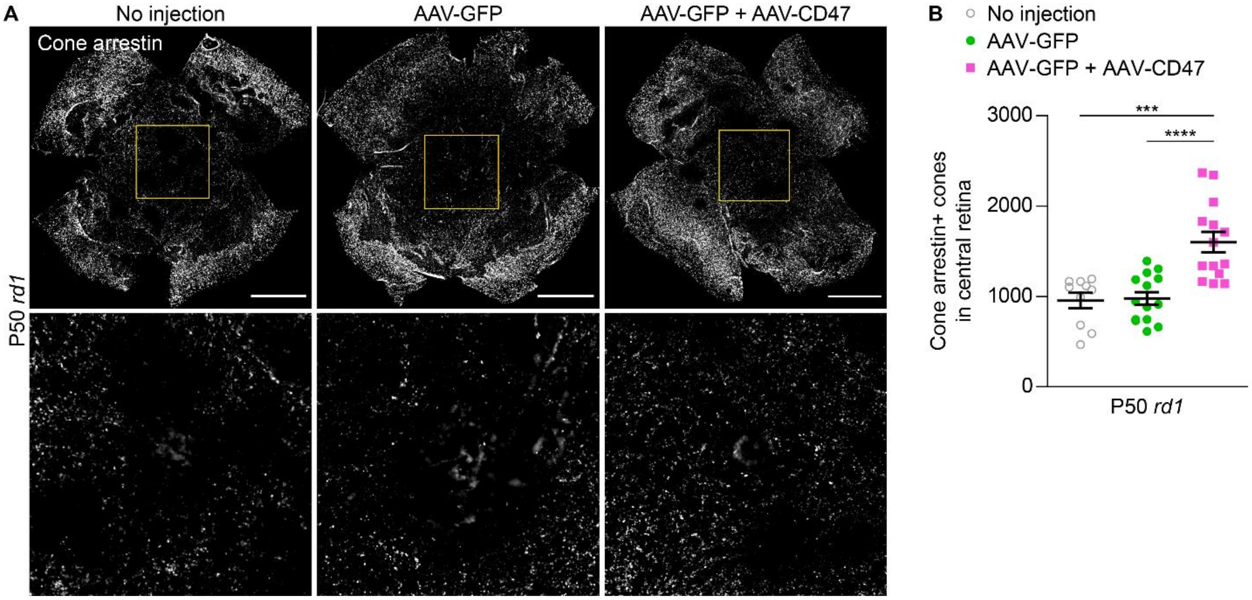

Supplemental Figure 3. Quantification of cone survival by immunostaining.

(**A**) Representative flat-mounts of P50 *rd1* retinas without treatment or following infection with AAV8-RedO-GFP or AAV8-RedO-GFP plus AAV8-RedO-CD47 after cone arrestin immunostaining. Paired images depict low and high magnifications. Scale bars, 1 mm. (**B**) Quantification of cone arrestin immunostaining in central retinas of *rd1* mice (n = 10-14) without treatment or following infection with AAV8-RedO-GFP or AAV8-RedO-GFP plus AAV8-RedO-CD47. Data are shown as mean ± SEM. *** P<0.001, **** P<0.0001 by two-tailed Student’s t-test.

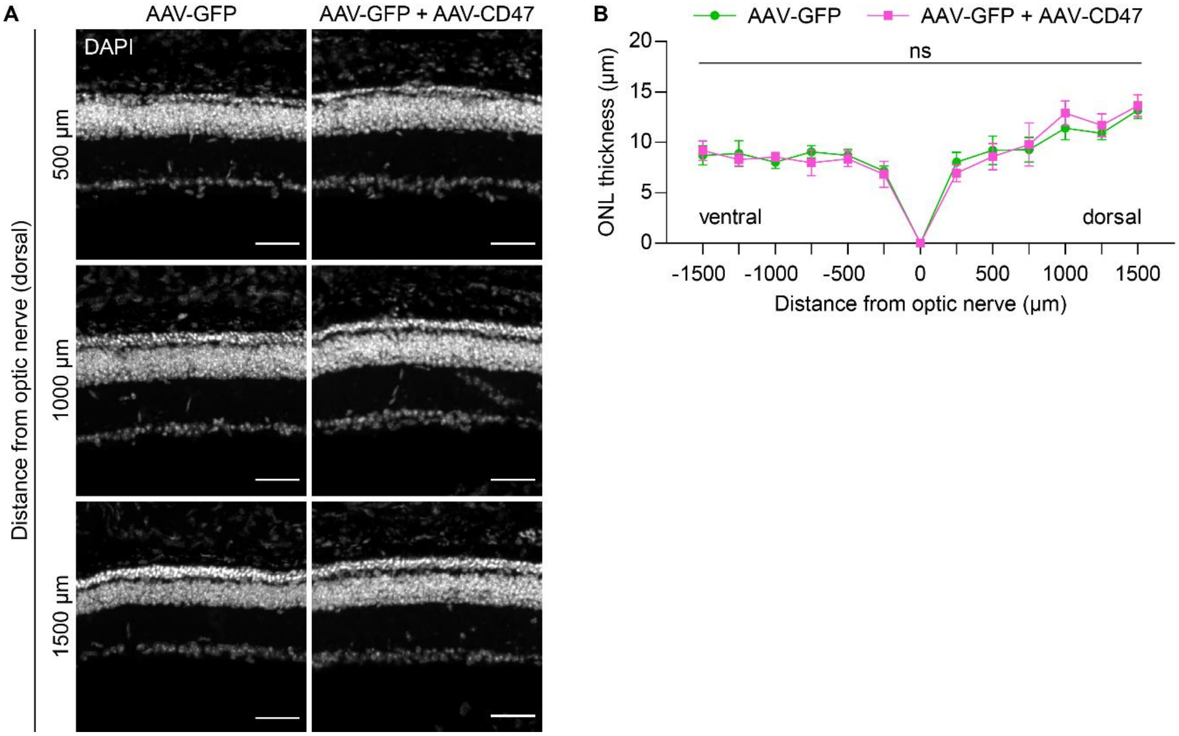

Supplemental Figure 4. Effect of CD47 expression on rod survival.

(**A**, **B**) Representative cross-sections (A) and measurements of outer nuclear layer (ONL) thickness (B) at indicated distances from the optic nerve in P40 *rd10* retinas (n = 5) following infection with AAV8-RedO-GFP or AAV8-RedO-GFP plus AAV8-RedO-CD47. Nuclei were labeled with DAPI. Scale bars, 50 µm. Data are shown as mean ± SEM. ns, not significant.

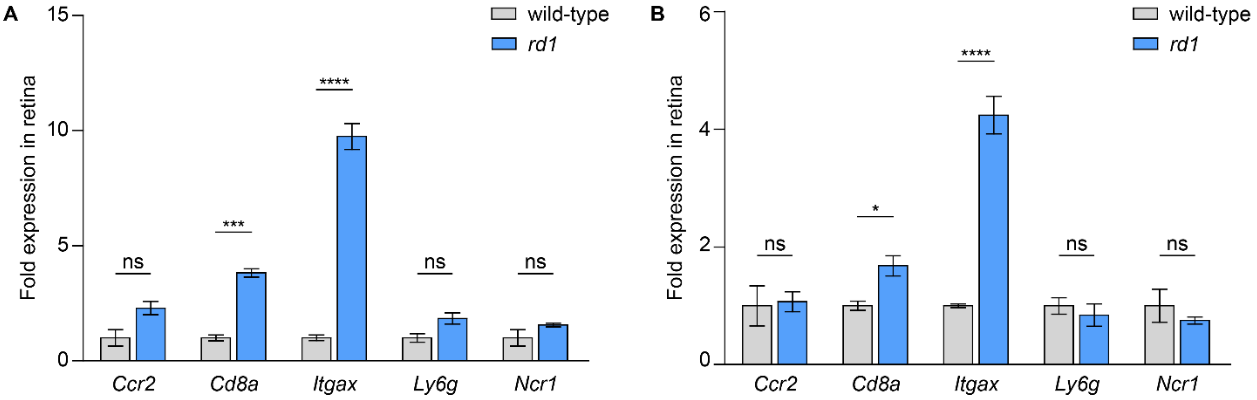

Supplemental Figure 5. Retinal expression of SIRPα-positive immune cell types.
(**A**, **B**) RNA expression of marker genes for monocytes (*Ccr2*), cytotoxic T cells (*Cd8a*), dendritic cells (*Itgax*), neutrophils (*Ly6g*), and natural killer cells (*Ncr1*) in retinas (n = 4–5) from 6- to 8-week-old wild-type (sighted FVB) or P40 *rd1* mice after normalization to either the housekeeping gene *Gapdh* (A) or the retinal ganglion cell marker *Thy1* (B). Data are shown as mean ± SEM. * $P<0.05$, *** $P<0.001$, **** $P<0.0001$ by two-tailed Student's t-test with Bonferroni correction. ns, not significant.

Supplemental Table 1. Summary of mouse experiments.

Mutation	Strain(s)	Assay or intervention	Age(s)
wild-type	Sighted CX3CR1$^{GFP/+}$	Ex vivo phagocytosis	P20, P50
	CD-1	Retinal histology	P40, P100
	CD-1	RPE histology	P100
	Sighted C3H	Light-dark discrimination	P30
	Sighted FVB	qPCR	6-8 weeks
rd1	*rd1*;CX3CR1$^{GFP/+}$	Ex vivo phagocytosis	P20, P50
	FVB, *rd1*;CreERT2/+, *rd1*;TSP1$^{-/-}$, *rd1*;SIRPα$^{-/-}$	Cone survival (histology)	P50
	FVB	Cone survival (flow cytometry)	P50
	C3H	Light-dark discrimination	P30
	rd1;CreERT2/+	Tamoxifen	P19-21
	FVB	Microglia depletion	P20-49
	rd1;TSP1$^{+/-}$, *rd1*;TSP1$^{-/-}$, *rd1*;SIRPα$^{+/-}$, *rd1*;SIRPα$^{-/-}$	Retinal histology	P30
	FVB	qPCR	P40
rd10	*rd10*	Cone survival (histology)	P130
	rd10	Cone survival (flow cytometry)	P100
	rd10	Rod survival	P40
	rd10	Optomotor	P60
Rho$^{-/-}$	*Rho*$^{-/-}$	Cone survival (histology)	P150

Supplemental Table 2. qPCR primer sequences.

Gene	5’	3’
Ccr2	ATCCACGGCATACTATCAACATC	CAAGGCTCACCATCATCGTAG
Cd8a	CCGTTGACCCGCTTTCTGT	CGGCGTCCATTTTCTTTGGAA
Gapdh	AGGTCGGTGTGAACGGATTTG	TGTAGACCATGTAGTTGAGGTCA
Itgax	CTGGATAGCCTTTCTTCTGCTG	GCACACTGTGTCCGAACTCA
Ly6g	GACTTCCTGCAACACAACTACC	ACAGCATTACCAGTGATCTCAGT
Ncr1	ATGCTGCCAACACTCACTG	GATGTTCACCGAGTTTCCATTTG
Thy1	TGCTCTCAGTCTTGCAGGTG	TGGATGGAGTTATCCTTGGTGTT
